# Prevalence, virulence profiling, and latent antimicrobial resistance determinants of *Escherichia coli* O157:H7 in imported Australian beef cattle during quarantine in Indonesia

**DOI:** 10.14202/vetworld.2026.2450-2462

**Published:** 2026-06-13

**Authors:** Siti Rakhma Afriana, Widagdo Sri Nugroho, Widodo Suwito

**Affiliations:** 1 1Department of Veterinary Public Health, Faculty of Veterinary Medicine, Universitas Gadjah Mada, Sleman 55281, Yogyakarta, Indonesia; 2 Research Center for Food Technology and Processing, National Research and Innovation Agency, Gunungkidul 55861, Yogyakarta, Indonesia

**Keywords:** antimicrobial resistance genes, biosecurity surveillance, genotype–phenotype discordance, imported beef cattle, *Escherichia coli* O157:H7, quarantine facilities, shiga toxin-producing *Escherichia coli*, zoonotic pathogen

## Abstract

**Background and Aim:**

*Escherichia coli* O157:H7 is an important zoonotic foodborne pathogen associated with severe gastrointestinal disease in humans and is increasingly recognized as a reservoir of antimicrobial resistance (AMR) determinants in food-producing animals. Imported beef cattle may contribute to the transboundary dissemination of pathogenic and antimicrobial-resistant bacteria during international livestock trade. This study aimed to determine the prevalence, virulence profiles, phenotypic antimicrobial susceptibility, and AMR genes of *E. coli* O157:H7 isolated from imported Australian beef cattle during quarantine in Indonesia.

**Materials and Methods:**

A cross-sectional study was conducted from March to November 2025 using 680 samples collected from imported beef cattle at five quarantine facilities in West Java and Banten, Indonesia. Samples included 650 rectal fecal specimens and 30 environmental samples. Isolation and identification of *E. coli* O157:H7 were performed using selective culture, biochemical characterization, and multiplex polymerase chain reaction targeting *rfbO157*, *fliCH7*, *stx1*, and *stx2*. Antimicrobial susceptibility testing was conducted using the Kirby–Bauer disk diffusion method. Detection of AMR genes (*tetA*, *strA*, *sul2*, *qnrS*, and *bla*_TEM_) was performed by polymerase chain reaction.

**Results:**

Of the 680 samples examined, 16 (2.35%) were confirmed as *E. coli* O157:H7, all originating from fecal samples. Among these isolates, 11 (68.75%) carried *stx1* and/or *stx2* genes and were classified as Shiga toxin-producing *E. coli*, whereas 5 (31.25%) were non-Shiga toxin-producing *E. coli* isolates. Antimicrobial susceptibility testing showed that most isolates remained susceptible to the tested antimicrobials. Resistance was highest to tetracycline (31.25%), followed by ampicillin (6.25%). No resistance was observed to streptomycin, trimethoprim–sulfamethoxazole, or enrofloxacin. Molecular analysis revealed the presence of AMR genes, including *tetA* (43.75%), *strA* (37.5%), *sul2* (18.75%), *qnrS* (18.75%), and *bla*_TEM_ (12.5%). Several isolates carried multiple resistance genes despite showing phenotypic susceptibility.

**Conclusion:**

Imported beef cattle during quarantine in Indonesia carried *E. coli* O157:H7 harboring virulence-associated Shiga toxin genes and latent AMR determinants. The presence of resistance genes in phenotypically susceptible isolates highlights the importance of integrating molecular surveillance with routine phenotypic testing to strengthen AMR monitoring and biosecurity measures within international live animal trade systems.

## INTRODUCTION

*Escherichia coli* O157:H7 is a zoonotic foodborne pathogen of major public health importance. Human infection with *E. coli* O157:H7 can cause severe gastrointestinal diseases, including hemorrhagic colitis and hemolytic uremic syndrome, thereby posing a substantial global food safety threat [1]. In addition to its virulence potential, *E. coli* O157:H7 has increasingly been recognized as a reservoir of antimicrobial resistance (AMR) determinants [2, 3]. The epidemiological significance of AMR-associated *E. coli* O157:H7 lies in its capacity to harbor and disseminate resistance genes via horizontal gene transfer, thereby facilitating the spread of resistance determinants among bacterial populations in animal production systems and the surrounding environment [2, 4].

Indonesia relies heavily on imported beef cattle to meet the growing domestic demand for meat, with Australia as one of the primary exporters [5]. During importation, cattle are temporarily housed in designated quarantine facilities, where inspection, observation, and biosecurity procedures are implemented before distribution, as regulated by the Indonesian Quarantine Law [6]. Previous surveillance studies conducted in Australia reported that the prevalence of *E. coli* O157:H7 in beef cattle ranges from approximately 1%–13%, depending on geographical location, management system, season, and sampling strategy [7–9]. Extensive surveillance has been performed within domestic cattle production systems in exporting countries such as Australia. However, information on the carriage of *E. coli* O157:H7 and associated AMR determinants during importation and quarantine in importing countries remains limited, particularly in regions with high volumes of live cattle imports, including Southeast Asia and the Middle East.

Indonesia represents one of the largest destinations for Australian live cattle imports, and quarantine facilities constitute a critical control point for preventing the introduction and dissemination of zoonotic pathogens and AMR determinants through international livestock trade. Monitoring AMR in imported cattle is essential not only for animal health management but also for environmental and public health protection. Livestock-associated bacteria carrying AMR determinants may disseminate resistance genes into the surrounding environment through fecal contamination, wastewater, and farm residues, thereby facilitating the circulation of resistance determinants among animal, human, and environmental microbial communities within a One Health framework [2, 10, 11]. Therefore, surveillance of AMR during quarantine is essential for strengthening biosecurity systems and minimizing the environmental dissemination of AMR determinants [4].

Several studies have reported discrepancies between genotypic and phenotypic AMR profiles in *E. coli* isolates recovered from livestock populations [2, 12, 13]. The detection of resistance genes in phenotypically susceptible isolates suggests the presence of latent resistance determinants that may remain undetected by conventional susceptibility testing. Such genotype–phenotype discordance highlights the importance of integrating molecular approaches into quarantine surveillance systems to improve the early detection of silent AMR reservoirs and transferable resistance determinants associated with international animal movement.

Cattle are recognized as important reservoirs of *E. coli* O157:H7 and may contribute to the environmental dissemination of AMR determinants [14, 15]. Nevertheless, studies specifically investigating the prevalence, virulence-associated Shiga toxin genes, phenotypic antimicrobial susceptibility, and latent AMR determinants of *E. coli* O157:H7 in imported cattle during quarantine in Indonesia remain scarce. Furthermore, limited information is available regarding the potential role of quarantine facilities as epidemiological interfaces for the introduction and dissemination of zoonotic pathogens and AMR determinants into importing countries. Most previous studies have focused on domestic cattle production systems in exporting countries, whereas data from the import–quarantine stage of international livestock trade remain limited. Consequently, the epidemiological significance of imported cattle as potential carriers of latent AMR determinants during quarantine has not been adequately elucidated.

Therefore, this study was conducted to determine the prevalence of *E. coli* O157:H7 in imported Australian beef cattle during quarantine in Indonesia and to characterize the virulence profiles, phenotypic antimicrobial susceptibility patterns, and selected AMR genes among the recovered isolates. In addition, this study aimed to evaluate the occurrence of genotype–phenotype discordance associated with AMR among the isolates recovered during quarantine. The findings of this study are expected to provide baseline epidemiological data to support molecular-based AMR surveillance, reinforcement of quarantine biosecurity, and risk assessment strategies for international live animal trade from a One Health perspective.

## MATERIALS AND METHODS

### Ethical approval

The study protocol involving imported beef cattle was reviewed and approved by the Research Ethics Committee of the Faculty of Veterinary Medicine, Universitas Gadjah Mada, Yogyakarta, Indonesia (Approval No. 28/EC-FKH/int./2025; approved on May 7, 2025). All sampling procedures were conducted in accordance with institutional ethical standards and national regulations governing animal handling and quarantine procedures in Indonesia. Rectal fecal sampling and environmental specimen collection were performed by trained personnel under veterinary supervision to minimize animal stress and avoid unnecessary discomfort. The study also adhered to the ARRIVE 2.0 guidelines for reporting *in vivo* animal research and was adapted to the operational procedures applicable to quarantine sampling of imported livestock in Indonesia.

### Study period and location

**Figure 1:**
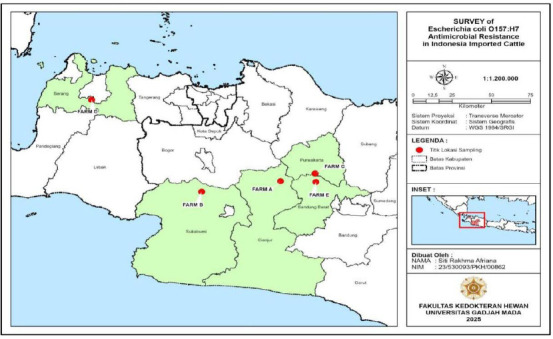
Geographic distribution of the five quarantine facilities included in this study located in West Java and Banten Provinces, Indonesia, used for sampling imported Australian beef cattle during quarantine. The map was generated using ArcGIS Pro version 3.3.2 (Esri, Redlands, CA, USA).

This study was conducted from March to November 2025 at five quarantine facilities under the jurisdiction of the DKI Jakarta Quarantine Office in Indonesia, where imported Australian beef cattle were temporarily housed during mandatory quarantine before distribution. The study locations included facilities A (Cianjur, West Java), B (Sukabumi, West Java), C (Purwakarta, West Java), D (Serang, Banten), and E (Bandung Barat, West Java). These quarantine facilities were selected because they actively received consignments of imported beef cattle during the study period and served as major receiving points within Indonesia’s live cattle importation system. The geographic distribution of the study locations is shown in Figure 1, and the approximate geographic coordinates of each facility are provided in Supplementary Table S1. Laboratory analyses were conducted at the Veterinary Public Health Laboratory, Faculty of Veterinary Medicine, Universitas Gadjah Mada, Yogyakarta, Indonesia.

### Study design

A cross-sectional study design was used to investigate the prevalence, virulence characteristics, phenotypic antimicrobial susceptibility, and determinants of AMR in *E. coli* O157:H7 isolated from imported Australian beef cattle during quarantine in Indonesia. Both fecal and environmental specimens were collected to evaluate the potential role of imported cattle as reservoirs of zoonotic pathogens and AMR determinants and to assess possible environmental contamination within quarantine facilities.

### Sample size determination

Sample collection was performed during the mandatory quarantine period immediately after cattle arrival at the quarantine facilities. The minimum required sample size for fecal sampling was calculated using the formula:


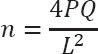


where P represents the expected apparent prevalence, Q = 1 − P, and L represents the desired absolute precision [16]. The expected apparent prevalence of Shiga toxin-producing *E. coli* (STEC) O157 in young Australian beef cattle was based on a national Australian survey reporting a prevalence of 8.4% [8]. The prevalence value was rounded to 9% (P = 0.09), resulting in Q = 0.91. Using a 95% confidence level and a desired precision of 5%, the calculated minimum sample size was 130 fecal samples. To improve representativeness and minimize sampling bias among quarantine facilities, the calculated sample size was multiplied by five [16], resulting in a target sample size of 650 fecal samples.

In the present study, fecal samples were collected from cattle distributed across five quarantine facilities, with 130 samples collected from each facility. In addition, six environmental samples were collected from each facility, resulting in a total of 680 samples included in this study.

### Sample collection

A total of 680 samples were collected, comprising 650 rectal fecal specimens from imported Australian beef cattle and 30 environmental specimens, including soil (n = 20), drinking water (n = 5), and wastewater (n = 5). Fecal specimens (approximately 50 g) were collected rectally from individual cattle using sterile disposable gloves. Soil specimens (approximately 50 g) were collected from the corners of cattle pens, whereas drinking water and wastewater specimens (100 mL) were collected from drinking troughs and wastewater holding ponds within the quarantine facilities using sterile conical tubes.

All samples were transported to the laboratory in cool boxes containing ice packs and processed immediately upon arrival. Environmental sampling was incorporated to evaluate potential contamination associated with imported cattle during quarantine and to assess possible spillover pathways of zoonotic bacteria and AMR determinants within live-animal import systems.

### Isolation and conventional identification of *E. coli*

Sample preparation was performed by homogenizing 10 g of fecal and soil specimens with 90 mL of Buffered Peptone Water (BPW; Oxoid CM0509, Oxoid, Hampshire, UK) using a stomacher. For water samples, an initial filtration step was performed using a 0.45 µm mixed cellulose membrane filter (Millipore, Merck Millipore, Darmstadt, Germany). Subsequently, 10 mL of the filtrate was transferred into 90 mL of BPW for enrichment. The homogenized samples were incubated at 37 ± 1°C for 18–24 h.

After enrichment, a loopful of culture was streaked onto Eosin Methylene Blue agar (EMB agar; Oxoid CM0069B, Oxoid, Hampshire, UK) to identify lactose-fermenting *E. coli* based on the characteristic green metallic sheen produced by acid precipitation of EMB dyes. A single well-isolated colony exhibiting a metallic sheen was selected and subjected to Gram staining. Microscopic examination confirmed Gram-negative short rod-shaped bacilli arranged singly or in pairs, consistent with the classical morphology of *E. coli* [17, 18]. When mixed colonies were observed, isolates were re-streaked to obtain pure cultures before further identification. Confirmed Gram-negative colonies were subsequently subcultured onto Nutrient Agar and subjected to standard biochemical identification procedures.

Biochemical confirmation included Indole medium (HiMedia M463-500G, HiMedia Laboratories, Mumbai, India), methyl red and Voges–Proskauer medium (HiMedia GM070I-500G), citrate medium (HiMedia M069-500G), and Triple Sugar Iron Agar (TSIA; Oxoid CM0277B, Oxoid, Hampshire, UK). Isolates showing an Indole, methyl red, Voges–Proskauer, and citrate (IMViC) pattern of (+ + − −) and TSIA reactions characterized by acid slant/acid butt with gas production and absence of hydrogen sulfide were classified as *E. coli*. This biochemical profile is consistent with established identification standards in veterinary microbiology [18, 19]. The reference strain *E. coli* O157:H7 American Type Culture Collection (ATCC) 35150 was used as a positive control throughout the isolation procedures, including EMB agar (Oxoid CM0069B, Oxoid, Hampshire, UK) and Sorbitol-MacConkey agar (SMAC agar; HiMedia M298-500G,) culture, to validate the expected morphological and biochemical characteristics.

### Presumptive identification of *E. coli* O157

Biochemically confirmed *E. coli* isolates were screened on SMAC agar (HiMedia M298-500G) to identify presumptive *E. coli* O157 isolates. Non-sorbitol-fermenting (NSF) colonies that appeared colorless or pale after incubation at 37°C for 24 h were classified as presumptive O157 isolates according to established diagnostic criteria [3, 20]. However, SMAC agar has limited specificity for *E. coli* O157:H7 because certain non-O157 STEC strains and other members of Enterobacteriaceae may also produce sorbitol-negative colonies, whereas some *E. coli* O157 strains may demonstrate variability in sorbitol fermentation [19, 21]. Therefore, molecular confirmation using polymerase chain reaction (PCR) targeting *rfbO157* and *fliCH7* genes was performed to accurately identify *E. coli* O157:H7 isolates.

### Molecular confirmation using multiplex PCR

Genomic DNA from all presumptive *E. coli* O157 isolates was extracted using the Quick-DNA™ Miniprep Plus Kit (Zymo Research, Irvine, CA, USA) according to the manufacturer’s instructions. Multiplex PCR assays were performed to confirm the *E. coli* O157:H7 serotype and determine the presence of virulence-associated genes. Four genetic targets were analyzed, including *rfbO157* (O157 somatic antigen), *fliCH7* (H7 flagellar antigen), and the Shiga toxin genes *stx1* and *stx2*. Primer sequences and expected amplicon sizes are presented in Table 1 [22–24].

**Table 1:** Primer sequences and expected amplicon sizes used for multiplex polymerase chain reaction.

Target gene	Primer sequence (5′–3′)	Amplicon size (bp)
*rfbO157*	F: CGG ACA TCC ATG TGA TAT GG	259
	R: TTG CCT ATG TAC AGC TAA TCC	
*fliCH7*	F: GCG CTG TCG AGT TCT ATC GAGC	625
	R: CAA CGG TGA CTT TAT CGC CAT TCC	
*stx1*	F: CGT CTT TAC TGA TGA TTG ATA GTG GC	637
	R: CGC GAT GCA TGA TGA TGA C	
*stx2*	F: TAC CAC TCT GCA ACG TGT CG	297
	R: CGA TAC TCC GGA AGC ACA TT	

The multiplex PCR assay targeted *rfbO157* and *fliCH7* for serotype confirmation and *stx1* and *stx2* for differentiation of STEC *E. coli* O157:H7 strains, thereby enabling assessment of the virulence potential of isolates recovered from imported cattle and providing epidemiological information relevant for risk-based quarantine surveillance.

Each PCR reaction was prepared in a total volume of 50 μL consisting of 25 μL PowerPol 2× PCR Master Mix (ABclonal Technology, Wuhan, China), 1 μL of each primer pair, 19 μL nuclease-free water, and 2 μL genomic DNA template. PCR amplification was performed using an initial denaturation step at 98°C for 45 s, followed by 30 cycles of denaturation at 98°C for 10 s, annealing at 57°C for 30 s, and extension at 72°C for 30 s, with a final extension at 72°C for 5 min.

PCR products were electrophoresed on 1.5% agarose gels stained with SYBR® Safe DNA Gel Stain (Invitrogen, Thermo Fisher Scientific, Waltham, MA, USA) and visualized under ultraviolet transillumination. Isolates showing simultaneous amplification of *rfbO157* (259 bp) and *fliCH7* (625 bp) were confirmed as *E. coli* O157:H7. The presence of *stx1* (637 bp) and *stx2* (297 bp) indicated STEC strains, whereas isolates lacking both toxin genes were classified as non-STEC *E. coli* O157:H7. The reference strain *E. coli* O157:H7 ATCC 35150 was included as a positive control in all PCR assays. A non-template control (NTC) containing all PCR components except DNA template was included as a negative control to monitor contamination and nonspecific amplification.

### Antimicrobial susceptibility testing

Antimicrobial susceptibility testing was performed using the Kirby–Bauer disk diffusion method on Mueller–Hinton agar in accordance with the Clinical and Laboratory Standards Institute (CLSI) guidelines [25]. The antimicrobial agents tested included ampicillin (10 µg), tetracycline (30 µg), enrofloxacin (5 µg), streptomycin (10 µg), and trimethoprim–sulfamethoxazole (1.25/23.75 µg). Bacterial suspensions were prepared and adjusted to a 0.5 McFarland standard before inoculation onto Mueller–Hinton agar plates. Antibiotic disks were placed on the inoculated agar surfaces, then incubated at 37°C for 18–24 h.

After incubation, inhibition zone diameters were measured in millimeters and interpreted as susceptible, intermediate, or resistant [25]. The interpretive criteria used for each antimicrobial agent are presented in Table 2. Each isolate was tested in duplicate to ensure reproducibility, and the reported inhibition zone diameter for each antimicrobial represented the mean value of duplicate measurements. Interpretations were based on CLSI M100 Performance Standards for Antimicrobial Susceptibility Testing, 35th edition, for Enterobacterales.

**Table 2:** Interpretation standards for inhibition zone diameters used in the Kirby–Bauer disk diffusion method [25].

No.	Antibiotic	Dose	Sensitive (S) (mm)	Intermediate (I) (mm)	Resistant (R) (mm)
1	Ampicillin	10 µg	≥17	14–16	≤13
2	Tetracycline	30 µg	≥15	12–14	≤11
3	Streptomycin	10 µg	≥15	12–14	≤11
4	Trimethoprim–sulfamethoxazole	1.25/23.75 µg	≥16	11–15	≤10
5	Enrofloxacin	5 µg	≥22	17–21	≤16

### Detection of AMR genes

AMR genes were detected by PCR to identify resistance determinants associated with the antimicrobial agents evaluated in this study. The selected genes represented major resistance determinants frequently associated with mobile genetic elements in livestock-associated *E. coli*, enabling identification of latent resistance potential even among phenotypically susceptible isolates. The target genes included β-lactam resistance gene *blaTEM*, tetracycline resistance gene *tetA*, aminoglycoside resistance gene *strA*, sulfonamide resistance gene *sul2*, and quinolone resistance gene *qnrS*. These genes were selected based on antimicrobial classes commonly used in livestock production and frequently reported among *E. coli* isolates recovered from Australian cattle populations [26, 27]. In addition, quinolone resistance genes were included because fluoroquinolones are categorized as critically important antimicrobials in human medicine [2, 28].

The same genomic DNA extracted using the Quick-DNA™ Miniprep Plus Kit (Zymo Research, Irvine, CA, USA) was used as the template for PCR amplification of AMR genes. Primer sequences, expected amplicon sizes, and annealing temperatures are presented in Table 3 [29–31]. PCR reactions were prepared in a total volume of 25 μL consisting of 2 μL genomic DNA template, 12.5 μL PowerPol 2× PCR Mix with Dye V2 (ABclonal Technology, Wuhan, China), 0.5 μL forward primer (10 μM), 0.5 μL reverse primer (10 μM), and 9.5 μL nuclease-free water.

**Table 3:** Primer sequences and annealing temperatures used for antimicrobial resistance genes.

Antibiotic class	Gene	Primer sequence (5′–3′)	Amplicon size (bp)	Annealing temperature (°C)	Reference
Penicillin	*blaTEM*	F: GCGGAACCCCTATTTG	963	52	[29]
		R: ACCAATGCTTAATCAGTGAG			
Tetracycline	*tetA*	F: GCTACATCCTGCTTGCCTTC	210	58	[30]
		R: CATAGATCGCCGTGAAGAGG			
Aminoglycoside	*strA*	F: TGGCAGGAGGAACAGGAGG	405	58	[31]
		R: AGGTCGATCAGACCCGTGC			
Sulfonamide	*sul2*	F: GGCAGATGTGATCGACCTCG	405	60	[30]
		R: ATGCCGGGATCAAGGACAAG			
Fluoroquinolone	*qnrS*	F: GCAAGTTCATTGAACAGGGT	428	54	[31]
		R: TCTAAACCGTCGAGTTCGGCG			

PCR amplification was performed using an initial denaturation step at 98°C for 45 s, followed by 30 cycles of denaturation at 98°C for 10 s, annealing at temperatures specific for each primer pair (Table 3), and extension at 72°C for 30 s, with a final extension step at 72°C for 5 min. PCR products were separated by agarose gel electrophoresis and visualized under ultraviolet transillumination. An NTC containing all PCR reagents except DNA template was included in each PCR run to monitor contamination. Amplicon sizes were verified using a DNA ladder during electrophoresis.

## RESULTS

### Isolation and identification of *E. coli*

A total of 680 samples were analyzed, comprising 650 fecal specimens and 30 environmental samples. Cultural and biochemical examination identified *E. coli* in 649 of 680 samples (95.44%), with the highest prevalence observed in fecal samples (96.15%; 625/650), indicating that feces constituted the primary source of *E. coli* detection in the sampled cattle population (Table 4). Colonies exhibiting a metallic green sheen on EMB agar were selected and confirmed as Gram-negative short rods through Gram staining. These isolates exhibited a characteristic *E. coli* biochemical profile, including Indole-positive, methyl red-positive, Voges–Proskauer-negative, and citrate-negative reactions, with an acid reaction on TSIA.

**Table 4:** Distribution of *Escherichia coli* and *E. coli* O157:H7 isolated from fecal samples collected from quarantine facilities.

Quarantine facility	n	*E. coli*	*E. coli* O157:H7	Shiga toxin-producing *E. coli* O157:H7	Non-STEC *E. coli* O157:H7
Facility A	130	125	2	2	0
Facility B	130	118	3	1	2
Facility C	130	128	7	6	1
Facility D	130	126	0	0	0
Facility E	130	128	4	2	2
Total	650	625 (96.15%)	16 (2.46%)	11 (1.69%)	5 (0.76%)

### Presumptive identification of *E. coli* O157

**Figure 2:**
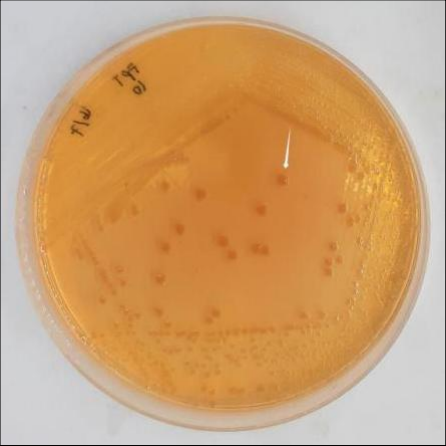
Presumptive identification of *Escherichia coli* O157 isolates on Sorbitol Mac Conkey agar showing non-sorbotol fermenting colonies appearing colorless or pale after incubation.

Biochemically confirmed *E. coli* isolates were screened on SMAC agar to identify NSF colonies. As shown in Figure 2, colorless colonies were presumptively identified as *E. coli* O157. Twenty-four isolates (3.8%) exhibited an NSF phenotype, all originating from fecal samples, whereas no environmental isolates showed the O157 phenotype at this stage.

### PCR confirmation of *E. coli* O157:H7

**Figure 3:**
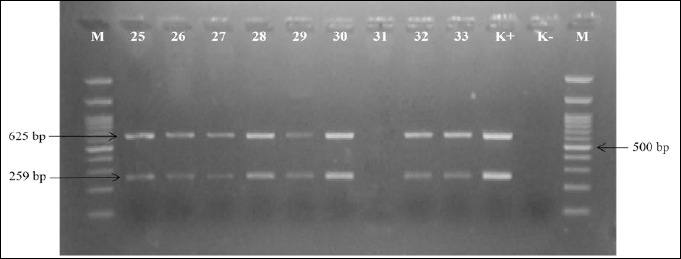
Multiplex polymerase chain reaction amplification of *Escherichia coli* O157:H7 targeting the *rfbO157* (259 bp) and *fliCH7* (625 bp) genes. Lanes 25, 26, 27, 28, 29, 30, 32, and 33 show positive amplification for both *rfbO157* and *fliCH7*, corresponding to sample codes FLP 42, FLP 62, FLP 63, FLP 78, FLP 103, FLP 117, FPT 9, and FCI 23, respectively. Lane M: 100 bp DNA ladder. Lane K+: Positive control (*E. coli* O157:H7 ATCC 35150). Lane K−: Non Template Control.

Multiplex PCR amplification targeting *rfbO157*, *fliCH7*, *stx1*, and *stx2* confirmed 16 of the 24 presumptive isolates as *E. coli* O157:H7. The distribution of confirmed isolates across quarantine facilities is summarized in Table 5. As illustrated in Figure 3, 16 isolates produced the expected O157 (259 bp) and H7 (625 bp) gene bands.

**Table 5:** Isolation and molecular identification of *Escherichia coli* O157:H7 from imported beef cattle in quarantine facilities.

Quarantine facility	Total samples	*E. coli*	*E. coli* O157:H7	Shiga toxin-producing *E. coli* (STEC) O157:H7	Non-STEC *E. coli* O157:H7
Facility A	136	130	2	2	0
Facility B	136	123	3	1	2
Facility C	136	132	7	6	1
Facility D	136	131	0	0	0
Facility E	136	133	4	2	2
Total	680	649	16	11	5

**Figure 4:**
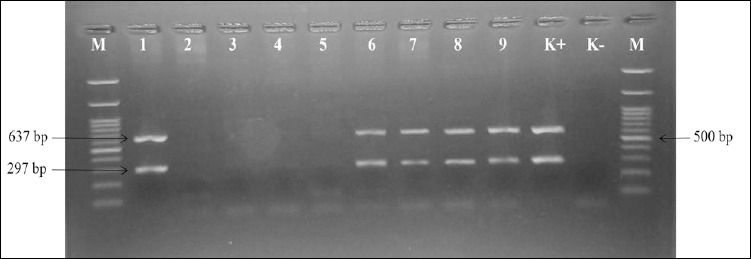
Multiplex Polymerase Chain Reaction amplification of *stx1* (637 bp) and *stx2* (297 bp) genes from *Escherichia coli* O157:H7 isolates. Positive amplification of Shiga toxin genes was observed in lanes 1, 6, 7, 8, 9, whereas all remaining lanes showed no amplification and were classified as negative for Shiga toxin genes. Lane M: 100 bp DNA ladder. Lane K+: Positive control (*E. coli* O157:H7 American Type Culture Collection 35150). Lane K−: Negative control.

Of the 16 confirmed *E. coli* O157:H7 isolates, 11 (68.75%) were classified as STEC *E. coli* O157:H7 carrying *stx1* and *stx2*, whereas 5 (31.25%) lacked both toxin genes and were classified as non-STEC *E. coli* O157:H7 (Table 5). Facility C showed the highest number of *E. coli* O157:H7 isolates (7/136), followed by facility E (4/136) and facility B (3/136), whereas no isolates were detected in facility D. The positive control (*E. coli* O157:H7 ATCC 35150) generated all target bands, whereas the NTC showed no amplification. Amplification patterns of the Shiga toxin genes are presented in Figure 4.

### Antimicrobial susceptibility testing

**Figure 5:**
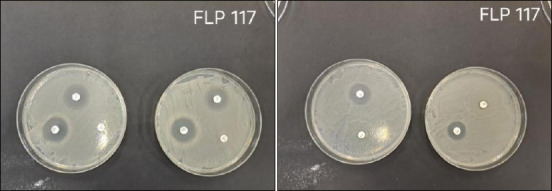
Representative antimicrobial susceptibility profile of *Escherichia coli* O157:H7 isolate FLP 117 determined using the Kirby–Bauer disk diffusion method, demonstrating resistance to tetracycline and ampicillin.

Antimicrobial susceptibility testing of the 16 *E. coli* O157:H7 isolates revealed variable resistance patterns across the five antibiotics tested (Table 6). Resistance was highest to tetracycline, with 5 of 16 isolates (31.25%) classified as resistant. Ampicillin resistance was detected in 1 isolate (6.25%). No resistance was observed to streptomycin, trimethoprim–sulfamethoxazole, or enrofloxacin. Intermediate susceptibility was observed for streptomycin in 3 isolates (18.75%), enrofloxacin in 2 isolates (12.5%), and ampicillin in 1 isolate (6.25%). All isolates were susceptible to trimethoprim–sulfamethoxazole. A representative antimicrobial susceptibility pattern is presented in Figure 5, illustrating isolate FLP 117, which exhibited resistance to tetracycline and ampicillin.

**Table 6:** Antibiotic susceptibility and resistance genes of *Escherichia coli* O157:H7 isolates determined using the Kirby–Bauer disk diffusion method.

No.	Antibiotic	Total tested	Sensitive	Intermediate	Resistant	Resistance gene
1	Ampicillin	16	14	1	1	2
2	Tetracycline	16	11	0	5	7
3	Streptomycin	16	13	3	0	6
4	Trimethoprim–sulfamethoxazole	16	16	0	0	3
5	Enrofloxacin	16	14	2	0	3
	Total	80	68 (85%)	6 (7.5%)	6 (7.5%)	21 (26.25%)

Resistant isolates were detected in facilities A, C, and E. Tetracycline resistance was identified in isolates from facilities A, C, and E, whereas ampicillin resistance was detected in only one isolate from facility C. No resistant isolates were detected in facility B or facility D. Analysis of resistance patterns showed that 11 of 16 isolates (68.75%) were susceptible to all tested antibiotics. Four isolates (25%) were resistant to a single antibiotic (tetracycline), whereas one isolate (6.25%) was resistant to two antibiotics (ampicillin and tetracycline).

### Detection of AMR genes

**Figure 6:**
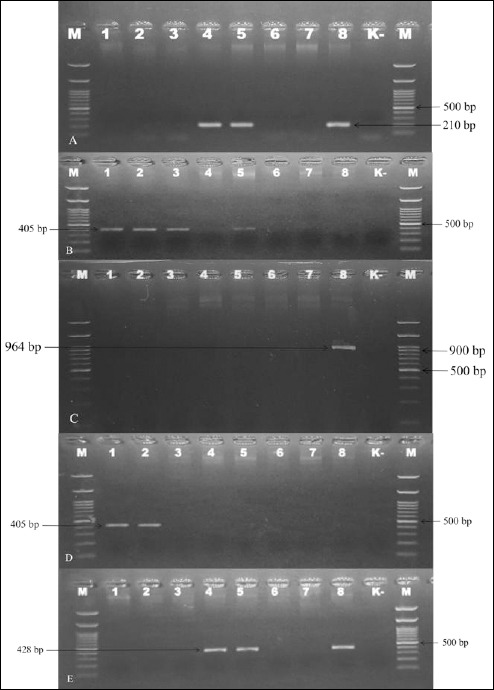
Representative Polymerase Chain Reaction amplification profiles of antimicrobial resistance genes detected in *Escherichia coli* O157:H7 isolates. (A) *tetA* (210 bp), (B) *strA* (405 bp), (C) *sul2* (405 bp), (D) *qnrS* (428 bp), and (E) *blaTEM* (963 bp). Lane M: 100 bp DNA ladder. Lane K−: Non-Template Control. Numbered lanes represent representative isolates showing positive amplification for the respective target genes.

PCR analysis revealed the presence of several AMR genes among the *E. coli* O157:H7 isolates (Table 4). The *tetA* gene was the most frequently detected, present in 7 of 16 isolates (43.75%), followed by *strA* in 6 isolates (37.5%). The *sul2* and *qnrS* genes were each detected in 3 isolates (18.75%), whereas *blaTEM* was detected in 2 isolates (12.5%). Representative amplification patterns of the resistance genes are shown in Figure 6.

Overall, eight isolates (50.0%) carried at least one AMR gene. Five isolates (31.25%) carried multiple resistance genes. The most common gene combinations were *tetA* + *strA* (n = 3), *tetA* + *strA* + *sul2* (n = 1), and *tetA* + *qnrS* (n = 1). Four isolates (25%) did not carry any of the resistance genes examined in this study. Although several resistance genes were detected, most isolates remained phenotypically susceptible to the corresponding antibiotics based on disk diffusion testing.

## DISCUSSION

### Prevalence of *E. coli* O157:H7 in imported cattle

This study provides the first characterization of *E. coli* O157:H7 virulence profiles and AMR genes in imported Australian beef cattle during quarantine in Indonesia. The overall prevalence of *E. coli* O157:H7 detected in this study was 2.35% (16/680), with 68.75% of confirmed isolates carrying *stx1* and/or *stx2*, indicating the presence of STEC among imported cattle entering the quarantine system. Importantly, AMR genes were detected in 50% of isolates despite predominantly susceptible phenotypes, suggesting the presence of latent AMR determinants in imported cattle populations.

The prevalence of *E. coli* O157:H7 observed in this study was relatively low compared with some reports from domestic cattle production systems. Based on fecal samples, the prevalence reached 2.46% (16/650), which falls within the range reported in surveys of Australian beef cattle populations. Previous studies have documented *E. coli* O157:H7 prevalence ranging from approximately 1%–13%, depending on feedlot management systems, geographic location, and sampling season [8, 9, 29]. More recent surveillance studies in Australian beef cattle similarly reported relatively low prevalence levels in most feedlot populations [9, 30]. Although the prevalence observed in the present study was at the lower end of this range, the detection of *E. coli* O157:H7 in clinically healthy imported cattle confirms that asymptomatic carriage remains common in bovine carriers and continues to represent a potential source of pathogen dissemination [15, 34].

### Distribution of STEC and non-STEC isolates

Multiplex PCR analysis revealed that 11 of the 16 confirmed *E. coli* O157:H7 isolates (68.75%) carried *stx1* and/or *stx2*, indicating the presence of toxigenic STEC strains. The detection of Shiga toxin genes is epidemiologically significant because these virulence determinants are strongly associated with severe human disease, including hemorrhagic colitis and hemolytic uremic syndrome [11, 32]. Similar patterns have been reported in Australian cattle populations, where the overall prevalence of *E. coli* O157:H7 is generally low, but detected isolates frequently harbor Shiga toxin genes, particularly *stx2*, which is associated with increased virulence [8, 9, 33].

In addition to STEC isolates, five strains (0.76%) were identified as non-STEC *E. coli* O157:H7, indicating the absence of both *stx1* and *stx2*. The coexistence of toxigenic and non-toxigenic variants is well documented in bovine populations and reflects the dynamic nature of *stx*-encoding bacteriophages. Because *stx* genes are carried by lysogenic bacteriophages, they may be gained or lost during bacterial evolution [14, 35]. Consequently, non-STEC *E. coli* O157:H7 strains remain epidemiologically relevant because they retain the *rfbO157* and *fliCH7* genetic markers and may reacquire Shiga toxin genes through horizontal phage transfer.

### Distribution among quarantine facilities

The distribution of *E. coli* O157:H7 varied among quarantine facilities, with the highest frequency observed in facility C (5.38%) and facility E (3.08%), whereas no isolates were detected in facility D. Heterogeneous distribution of *E. coli* O157:H7 among cattle groups has been widely reported and is often associated with differences in animal origin, management conditions, and environmental exposure during transport or holding [15, 34]. Such variability highlights the importance of facility-level surveillance in quarantine systems receiving imported cattle consignments.

The distribution of STEC and non-STEC *E. coli* O157:H7 isolates also varied across quarantine facilities. STEC *E. coli* O157:H7 were most frequently detected in facility C (6/7 isolates), whereas facilities B and E contained a mixture of STEC and non-STEC variants. Facility A contained only STEC *E. coli* O157:H7 (2/2), whereas no *E. coli* O157:H7 isolates were detected in facility D. These findings indicate heterogeneity in the distribution of toxigenic strains among imported cattle consignments. Such variation may reflect differences in the origins of cattle batches, transport conditions, or management practices prior to export. Previous studies have reported that STEC *E. coli* O157:H7 shedding in cattle populations is often clustered within specific groups or cohorts rather than evenly distributed across herds [15, 34].

### Environmental contamination and biosecurity implications

The results of environmental sampling from soil, wastewater, and drinking water collected from the quarantine facilities are presented in Table 7. Environmental sampling detected commensal *E. coli* in soil and wastewater, but *E. coli* O157:H7 was not detected in any environmental specimens (0/30). The detection of *E. coli* in soil and wastewater reflects the role of fecal shedding in contaminating the surrounding environment, a phenomenon widely reported in cattle production systems [2, 16]. However, the absence of *E. coli* O157:H7 in environmental samples may indicate limited environmental persistence or low shedding intensity during the sampling period.

**Table 7:** Environmental detection of *Escherichia coli* and *E. coli* O157:H7 from soil, wastewater, and drinking water samples collected from quarantine facilities.

Quarantine facility	Soil n	Soil *E. coli*	Soil *E. coli* O157:H7	Wastewater n	Wastewater *E. coli*	Wastewater *E. coli* O157:H7	Drinking water n	Drinking water *E. coli*	Drinking water *E. coli* O157:H7
Facility A	4	4	0	1	1	0	1	0	0
Facility B	4	4	0	1	1	0	1	0	0
Facility C	4	4	0	1	0	0	1	0	0
Facility D	4	4	0	1	1	0	1	0	0
Facility E	4	4	0	1	1	0	1	0	0
Total	20	20	0	5	4	0	5	0	0

### Phenotypic antimicrobial susceptibility patterns

Phenotypic antimicrobial susceptibility testing showed that most *E. coli* O157:H7 isolates remained susceptible to the tested antimicrobials, with resistance confined to a small subset of strains. Similar patterns of low AMR in cattle-derived *E. coli* O157:H7 have been reported in regions with strong antimicrobial stewardship policies [12, 26, 27]. Australian surveillance programs likewise reported relatively low rates of resistance among *E. coli* isolates from beef cattle [26, 27].

### Genotype–phenotype discordance of AMR determinants

Despite the largely susceptible phenotypic profile, molecular analysis revealed the presence of AMR genes including *tetA*, *strA*, *sul2*, *qnrS*, and *blaTEM*. These genes were detected in several isolates, even though phenotypic resistance was not always observed. Such genotype–phenotype discordance has been documented in livestock-associated *E. coli*, where resistance genes may persist on mobile genetic elements without conferring detectable resistance under routine susceptibility testing conditions [2, 12]. Discordance between genotypic detection and phenotypic susceptibility has been widely reported in *E. coli* from livestock and environmental sources and may result from low-level gene expression, absence of promoter elements, plasmid copy number variation, active efflux systems, or silent carriage on mobile genetic elements [12–14, 36]. These latent resistance determinants may remain undetected during routine susceptibility testing but may be expressed under selective antimicrobial pressure, thereby facilitating the horizontal dissemination of resistance genes within bacterial populations.

### Biosecurity significance of plasmid-mediated resistance genes

The detection of plasmid-mediated resistance genes such as *qnrS* and *blaTEM* in phenotypically susceptible isolates underscores a previously under-recognized risk in the live animal trade: the silent introduction of transferable resistance determinants into importing countries. This observation highlights a potential biosecurity concern associated with international live-animal trade and supports integrating molecular AMR screening into quarantine protocols, offering a novel recommendation for biosecurity in high-volume import nations such as Indonesia.

### Study novelty and One Health relevance

Although the prevalence observed in this study falls within the range reported in Australian cattle production systems [8], the novelty of this work lies in its focus on the import–quarantine stage of the international livestock trade pathway. Most previous studies investigated *E. coli* O157:H7 in domestic feedlot or slaughterhouse settings within exporting countries. In contrast, the present study evaluated pathogen carriage and AMR gene profiles during quarantine in the importing country, a critical point at which pathogens and AMR determinants may be introduced into new production environments. Therefore, surveillance at quarantine entry points provides valuable biosecurity insight for monitoring the transboundary dissemination of zoonotic pathogens and AMR within a One Health framework [2, 4].

### Study limitations and future perspectives

This study has several limitations. First, the number of confirmed *E. coli* O157:H7 isolates was relatively small (n = 16), which limited the ability to perform statistical comparisons among quarantine facilities. Second, plasmid replicon typing and conjugative transfer assays were not conducted, preventing detailed characterization of the genetic context of the detected resistance genes. Third, the study did not determine *stx* subtypes (e.g., *stx2a*, *stx2c*), which may influence virulence potential.

Despite these limitations, the present study provides novel data on virulence profiles and latent AMR genes in imported cattle during the quarantine phase of international livestock trade. Future studies involving longitudinal sampling during transport and post-distribution stages would further improve understanding of pathogen dynamics in the global beef supply chain.

## CONCLUSION

This study demonstrated that imported Australian beef cattle during quarantine in Indonesia carried *E. coli* O157:H7 harboring virulence-associated Shiga toxin genes and latent AMR determinants. The overall prevalence of *E. coli* O157:H7 was 2.35%, with most confirmed isolates classified as STEC carrying *stx1* and/or *stx2*. Although phenotypic AMR was generally low, molecular analysis revealed the presence of important resistance genes including *tetA*, *strA*, *sul2*, *qnrS*, and *bla*_TEM_, indicating genotype–phenotype discordance and the existence of silent AMR reservoirs within imported cattle populations.

The findings highlight the epidemiological significance of imported cattle as potential carriers of transferable AMR determinants, despite their predominantly susceptible antimicrobial profiles. The detection of plasmid-mediated resistance genes in phenotypically susceptible isolates emphasizes the limitation of relying solely on conventional susceptibility testing and supports the integration of molecular-based AMR surveillance into routine quarantine monitoring systems. From a practical perspective, strengthening molecular screening at quarantine entry points could improve early detection of zoonotic pathogens and latent resistance determinants, thereby supporting biosecurity, food safety, and One Health-based antimicrobial stewardship programs in importing countries.

A major strength of this study was the combined evaluation of prevalence, virulence-associated genes, phenotypic antimicrobial susceptibility, and molecular determinants of resistance during the quarantine stage of international livestock trade, a critical yet underexplored point in pathogen surveillance. In addition, the inclusion of both fecal and environmental sampling provided broader insight into the potential environmental dissemination of AMR determinants associated with imported cattle.

Overall, this study provides important baseline epidemiological data regarding *E. coli* O157:H7 and latent AMR determinants in imported cattle during quarantine in Indonesia. These findings reinforce the importance of integrated molecular and phenotypic surveillance approaches to strengthen quarantine biosecurity systems and mitigate the transboundary dissemination of zoonotic pathogens and AMR through global live animal trade.

## DATA AVAILABILITY

The data generated during the study are included in the manuscript.

## AUTHORS′ CONTRIBUTIONS

SRA, WSN, and WS: Conceptualization, study design, data analysis, and interpretation. SRA: Sample collection, laboratory analysis, and Writing – original draft preparation. WSN and WS: Supervision and critical revision of the manuscript. All authors have read and approved the final version of the manuscript.
